# Effects on outcome of patients with severe sepsis and septic shock admitted to the ICU after implementation cooperative sepsis management protocol

**DOI:** 10.1186/cc11791

**Published:** 2012-11-14

**Authors:** R Champunot, N Kamsawang, D Thimsri, P Tuandoung, S Tansuphaswasdikul

**Affiliations:** 1 Buddhachinaraj Phitsanulok Hospital, Phitsanulok, Thailand

## Background

The application in clinical practice of evidence-based guidelines for the cooperative management of patients with severe sepsis and septic shock between community hospitals and tertiary referral hospital is still poor. The purpose of this study was to examine the outcome of patients with severe sepsis and septic shock admitted to the ICU after implementation of a cooperative sepsis management protocol.

## Methods

**Figure 1:**
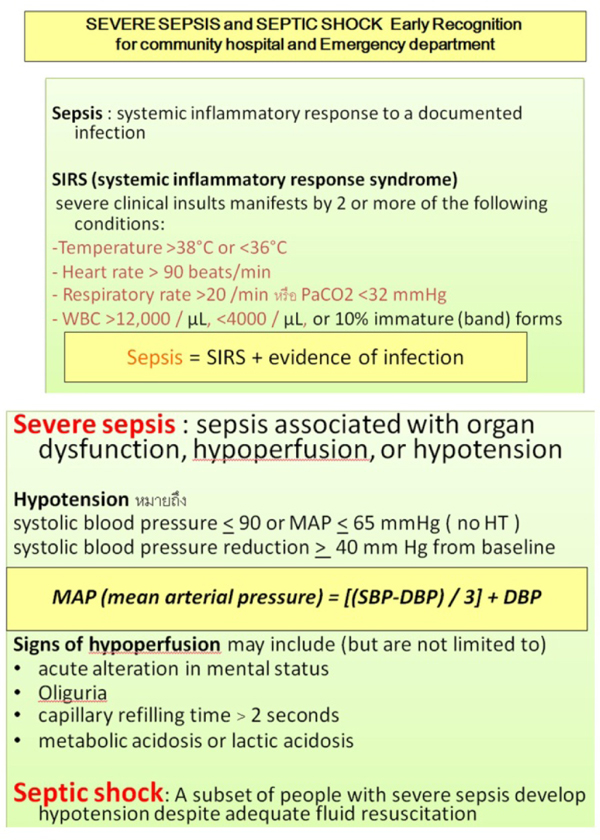
**Checklist for early recognition patients with severe sepsis and septic shock**.

**Figure 2:**
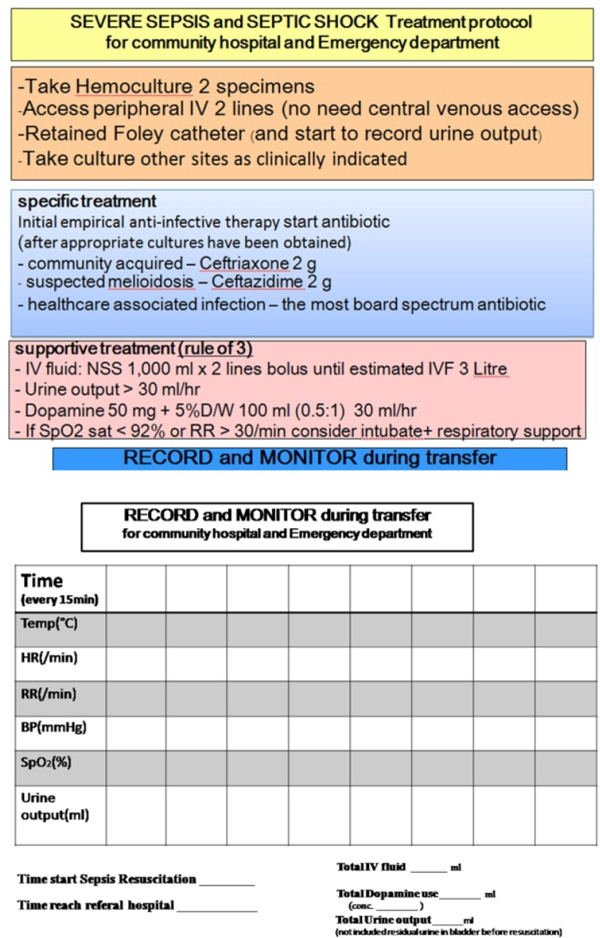
**Checklist for treatment and transport for severe sepsis and septic shock**.

From 1 October to 30 November 2009, patients with severe sepsis and septic shock admitted to the ICU received standard therapy (control group). From 1 December 2009 through 31 January 2010, patients with severe sepsis and septic shock (protocol group) were managed with a cooperative sepsis management protocol. The protocol included early recognition and the initiation of therapy by enabling nurses and physicians in community hospitals to mobilize institutional resources for the treatment of patients with severe sepsis and septic shock (Figures 1 and 2). Using goal-directed resuscitation protocols, early intensivist involvement and rapid transfer to the ICU from emergency department were implemented in tertiary referral hospital.

## Results

Sixty-two and 77 patients, respectively, were enrolled in the control and protocol groups. Rapid transfer of patients with severe sepsis and septic shock to the ICU from emergency department was observed in 1/62 (1.5%) of the control group and 45/77 (58.4%) of the protocol group (difference 56.9%; *P *= 0.01). The hospital mortality rate was 62.3% in the control group and 37.7% in the protocol group (*P *= 0.01). The protocol group had significant reductions in ICU length of stay, ICU cost and numbers of organ failure (*P *= 0.01).

## Conclusion

Empowerment of nurses and physicians in a community hospital to mobilize hospital resources for taking care of patients with severe sepsis and septic shock and implementation of a cooperative sepsis management protocol between community hospitals and tertiary referral hospital was temporally associated with improved outcomes.

